# Effect of Semaglutide on Thigh Skeletal Muscle Volume, Fat Infiltration and Physical Function in People With Obesity: Secondary Analysis of STEP UP

**DOI:** 10.1111/dom.71323

**Published:** 2026-09-20

**Authors:** Jøran Hjelmesæth, Suhas Bhat, W. Timothy Garvey, Kristoffer Jensen Kolnes, Jennifer Linge, Ildiko Lingvay, Niklas Kahr Rasmussen, Julio Rosenstock, Sean Wharton

**Affiliations:** ^1^ Department of Endocrinology, Obesity and Nutrition Vestfold Hospital Trust Tønsberg Norway; ^2^ Department of Endocrinology, Morbid Obesity and Preventive Medicine, Institute of Clinical Medicine University of Oslo Oslo Norway; ^3^ Novo Nordisk Global Business Services Bangalore India; ^4^ University of Alabama at Birmingham Birmingham Alabama USA; ^5^ Novo Nordisk A/S Søborg Denmark; ^6^ AMRA Medical AB Linköping Sweden; ^7^ Department of Health, Medicine and Caring Sciences Linköping University Linköping Sweden; ^8^ Department of Internal Medicine/Endocrinology and Peter O'Donnell Jr. School of Public Health University of Texas Southwestern Medical Center Dallas Texas USA; ^9^ University of Texas Southwestern Medical Center Dallas Texas USA; ^10^ University of Toronto Toronto Ontario Canada

**Keywords:** antiobesity drug, clinical trial, GLP‐1 analog, randomised trial, semaglutide, weight management

## Abstract

**Aims:**

To assess the effect of semaglutide on body composition and physical function.

**Materials and Methods:**

The phase 3b STEP UP trial randomised adults with body mass index ≥ 30 kg/m^2^ to once‐weekly subcutaneous semaglutide 7.2 mg, semaglutide 2.4 mg or placebo for 72 weeks. This prespecified secondary analysis assessed changes in MRI body composition and physical function.

**Results:**

Of 1407 participants, 55 with a valid baseline scan were included (semaglutide 7.2 mg, *n* = 43, 2.4 mg, *n* = 6 [pooled semaglutide group, *n* = 49]; placebo, *n* = 6). Estimated treatment differences (95% confidence intervals; *p*‐values) for absolute changes in adipose/muscle compartment volumes for the pooled semaglutide versus placebo group, respectively, were: −11.1 L (−16.5 to −5.7; *p* < 0.0001) for adipose body tissue, −1.5 L (−2.9 to −0.1; *p* = 0.04) for visceral adipose tissue, −1.7 L (−4.0 to 0.7; *p* = 0.16) for lean body tissue, −1.1 L (−2.3 to 0.1; *p* = 0.07) for thigh skeletal muscle volume and −0.92%‐points (−1.41 to −0.42; *p* = 0.001) for thigh muscle fat infiltration. Similar results were seen for relative changes in adipose/muscle compartment volumes. A sensitivity analysis for the semaglutide 7.2 mg group versus placebo showed similar results. A mean of 13 sit‐to‐stand repetitions in 30 s at baseline, and 15 repetitions at Week 72, was observed in the pooled semaglutide and placebo groups.

**Conclusions:**

Semaglutide plus lifestyle intervention was associated with reductions in adipose tissue volume, skeletal muscle volume and skeletal muscle fat infiltration, with a preserved number of sit‐to‐stand repetitions. Further research to assess the impact of weight loss on skeletal muscle composition and function should be explored in the future.

**Trial Registration:** ClinicalTrials.gov identifier NCT05646706

## 1 Introduction

Randomised clinical trials of glucagon‐like peptide‐1 (GLP‐1) and dual GLP‐1/glucose‐dependent insulinotropic polypeptide (GIP) receptor agonists (RAs), including semaglutide and tirzepatide, have demonstrated substantial and sustained bodyweight reduction, which is associated with reduced risk of developing obesity‐related complications and diseases, and improved quality of life [1]. Once‐weekly subcutaneous semaglutide 2.4 mg is approved for bodyweight management in people with overweight/obesity, and for risk reduction of major adverse cardiovascular events in people with overweight/obesity and established cardiovascular disease [2].

As with any weight‐loss intervention, such as bariatric surgery or dietary and pharmacological approaches, lean body mass is reduced along with fat mass [3, 4]. Estimates that up to 40% of the bodyweight loss induced by GLP‐1RA treatment may be from lean body mass (measured by dual‐energy X‐ray absorptiometry [DXA]) sparked questions around implications for skeletal muscle health, defined by muscle quantity, quality or function [5, 6, 7, 8, 9, 10]. Although lean body mass comprises muscle tissue, other organs and fluids, it is commonly conflated with skeletal muscle mass in the literature, confounding the interpretation of treatment effects on skeletal muscle health [11].

Skeletal muscle mass declines during weight loss, but some evidence suggests that this change may be adaptive and not a negative effect of weight loss on muscle health, especially when considering potential beneficial effects on muscle quality [5, 6, 8, 12]. In view of this, a recent publication highlighted various definitions of muscle quality in obesity, and, in addition, how changes in muscle quality can be assessed during weight loss in research and clinical practice settings [13]. Muscle quality may be defined by muscle composition (*morphological* muscle quality), which assesses fat infiltration into the muscle, or physical function (e.g., as measured by the sit‐to‐stand test). This is in contrast to muscle quantity, which comprises the overall volume or mass of muscle as measured using computed tomography or magnetic resonance imaging (MRI), or traditionally estimated through body composition assessments (e.g., DXA and ultrasound) [14]. There is thus a need to better understand how obesity medications, including GLP‐1 and dual GLP‐1/GIP RAs, affect body composition, and how reductions in adipose tissue and skeletal muscle mass are apportioned with bodyweight reductions after treatment with these drugs.

In the STEP UP trial, the magnitude of bodyweight reduction achieved using a higher 7.2 mg dose of semaglutide was superior to the currently available 2.4 mg dose and placebo in people with obesity [15]. MRI assessment was also included to allow in‐depth evaluations of body composition changes.

This prespecified secondary analysis of STEP UP focused on the subset of participants who underwent MRI scanning to provide an exploratory insight into, and to compare changes in, body composition accompanying bodyweight reduction. Specifically, the effects of semaglutide on thigh skeletal muscle volume and fat infiltration, quantified using MRI, and the number of sit‐to‐stand repetitions (as a marker of physical function) were assessed.

## 2 Methods

### 2.1 Study Design and Participants

STEP UP (ClinicalTrials.gov, NCT05646706) was a phase 3b, randomised, double‐blind, placebo‐controlled trial. The full trial design, including eligibility criteria, has been reported previously [15].

In brief, eligible participants were adults (aged ≥ 18 years) with obesity (body mass index [BMI] ≥ 30 kg/m^2^) and a history of at least one self‐reported unsuccessful dietary effort to lose bodyweight. Key exclusion criteria were a history of type 1 or type 2 diabetes, glycated haemoglobin ≥ 6.5% (≥ 48 mmol/mol) or a self‐reported change in bodyweight > 5 kg within 90 days before screening [15]. The trial was conducted in accordance with the Declaration of Helsinki and the International Conference on Harmonisation of Technical Requirements for Pharmaceuticals for Human Use Good Clinical Practice guidelines. Written informed consent was required before participating in the trial. The protocol for STEP UP has been published previously and can be accessed online [15].

This prespecified analysis of supportive secondary and exploratory endpoints focused on the STEP UP MRI subpopulation. Participants were enrolled in the MRI subpopulation at eligible sites if they had bodyweight < 149 kg at screening, no metal implants (e.g., pacemaker, intracranial clip, cochlear implant, infusion pump or neurostimulator) and were deemed suitable for MRI at the investigator's discretion (e.g., no claustrophobia).

### 2.2 Randomisation and Masking

In STEP UP, participants were randomly assigned (5:1:1) to receive once‐weekly subcutaneous semaglutide 7.2 mg, 2.4 mg or placebo, as an adjunct to lifestyle intervention comprising a reduced‐calorie diet (500 kcal deficit per day) and increased physical activity (at least 150 min of physical activity per week was encouraged, e.g., walking or using the stairs), for 72 weeks, followed by 9 weeks of off‐treatment follow‐up (additional details Supporting Information, p. 3).

### 2.3 Procedures

Participants in the subpopulation underwent body composition assessment using MRI at the beginning and end of treatment (two scans from the neck to the knees) to investigate the extent to which weight loss was caused by a reduction in adipose or lean body tissue volumes. The first scan was performed between screening and randomisation and the other within 7 days before or at the end of treatment at Week 72. MRI data were acquired as previously described (Supporting Information, p. 3–5) and body composition images were analysed by AMRA Researcher (AMRA Medical AB, Linköping, Sweden). All AMRA personnel were blinded to participants' treatment allocation throughout the whole image analysis process. An example of the body composition analysis in a participant is shown in the Supporting Information (p. 10, Figure S1). In addition, all participants underwent the sit‐to‐stand test at baseline and end of treatment.

### 2.4 Outcomes

MRI‐defined body composition changes were supportive secondary endpoints assessed as absolute and relative changes from baseline to Week 72 in: adipose and lean body tissue volumes (thoracic vertebra T9 to the knees, L, %); visceral adipose tissue volume (L, %); thigh skeletal muscle volume (L, %); thigh muscle fat infiltration (%‐points); and absolute changes in thigh skeletal muscle volume *z*‐score (number of standard deviations [SD]). MRI outcomes were obtained as previously described [16]. Supportive secondary endpoints in the MRI subpopulation included changes from baseline to Week 72 in bodyweight (kg).

Changes in thigh skeletal muscle volume and muscle fat infiltration (intramuscular fat) were exploratory endpoints. Muscle volume *z*‐scores (expressed as a number of SD) were calculated based on thigh skeletal muscle volume to provide sex‐, height‐, weight‐ and BMI‐invariant measures for each participant, expressed as the deviation from the mean value of matched individuals [17], with a score of zero indicating a skeletal muscle volume as expected, and negative or positive scores indicating lower or higher skeletal muscle volumes than expected, respectively [18].

The 30‐s sit‐to‐stand test was used as a surrogate measure of physical function. The test measured the change in absolute number of repetitions from baseline to Week 72 and was a prespecified exploratory endpoint of the trial. The sit‐to‐stand test has been shown to be a valid and highly reproducible assessment of muscle function for individuals with obesity [19, 20].

### 2.5 Statistical Analysis

Statistical methods for the STEP UP trial have been reported previously [15], and further details are provided in the Supporting Information (p. 6–7).

All data are from the in‐trial period, covering all on‐ or off‐treatment data points collected during the uninterrupted period from the date of randomisation until the last trial visit, regardless of discontinuation, dose reduction or initiation of other obesity therapies (including medications or bariatric surgery). All analyses used the treatment policy estimand, assessing treatment effect among all randomised participants, regardless of trial product discontinuation, dose reduction or initiation of any other obesity therapies or surgery. Data for the semaglutide 7.2 mg and 2.4 mg doses were pooled for analysis versus placebo, as prespecified in the primary trial statistical analysis plan. Additionally, a sensitivity analysis comparing semaglutide 7.2 mg with placebo was performed. Descriptive semaglutide 2.4 mg data are included in the Supporting Information (p. 11) for completeness. Owing to the limited sample sizes, notably in the placebo group, these prespecified analyses should be considered exploratory and hypothesis generating.

The estimated treatment differences (ETDs) in adipose and lean body tissue volumes were converted to ETDs in mass by multiplying the volumes by the corresponding tissue densities of 0.92 (adipose tissue) or 1.1 (lean tissue) [21]. Two‐sided 95% confidence intervals (CIs) were provided for all ETDs and within‐group changes, and a corresponding *p*‐value for ETDs.

The proportions of weight loss attributable to adipose and lean body tissue loss (from thoracic vertebra T9 to the knees) were calculated as detailed in the Supporting Information (p. 7).

All continuous endpoints were analysed with an analysis of covariance model (linear regression) with randomised treatment as a factor and the baseline value of the endpoint as a covariate. A multiple imputation approach was used to impute missing data 1000 times with assessments from retrieved participants in the placebo group, using a jump to reference approach. Statistical analyses were done with SAS software (version 9.4).

## 3 Results

The STEP UP trial included 1407 participants. This prespecified secondary analysis included 55 participants with a valid MRI scan at baseline who were randomly assigned to receive semaglutide 7.2 mg (*n* = 43/55), semaglutide 2.4 mg (*n* = 6/55) or placebo (*n* = 6/55) (Figure 1). At Week 72, MRI‐defined body composition data were available for 50 (90.9%) of 55 participants; 45 of 50 in the pooled semaglutide 7.2 mg/2.4 mg group and 5 of 50 in the placebo group. Most participants (52 [94.5%] of 55) completed the trial (attended end‐of‐trial visit at Week 81; 47 [95.9%] of 49 receiving pooled semaglutide and 5 [83.3%] of 6 receiving placebo). Similarly, the majority of participants completed the trial on‐treatment at Week 72 (45 [81.8%] of 55 in the overall MRI subpopulation; 40 [81.6%] of 49 receiving pooled semaglutide and 5 [83.3%] of 6 receiving placebo). All data below are reported for the STEP UP MRI subpopulation.

**FIGURE 1:**
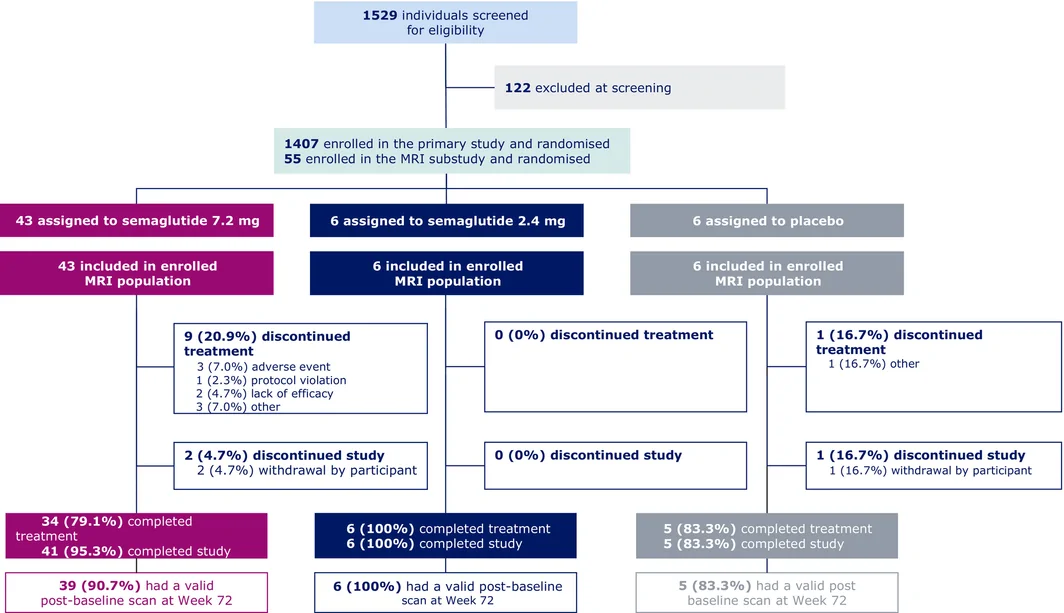
Trial profile—participant flow. MRI, magnetic resonance imaging.

Overall, baseline characteristics were generally well‐balanced across treatment groups (Table 1) in the MRI subpopulation, and in line with the total STEP UP population. Most participants were female (43 [78.2%] of 55) and White (47 [85.5%] of 55). The mean (SD) age was 45 (12) years, mean bodyweight was 110.8 (19.7) kg, and mean BMI was 39.3 (5.9) kg/m^2^. Overall, 21 (38.2%) of 55 participants had prediabetes. Baseline body composition, including mean adipose tissue volume, lean body tissue volume, visceral adipose tissue volume, thigh skeletal muscle volume, muscle volume *z*‐score and muscle fat infiltration (%), was similar between groups (Table 1).

**TABLE 1:** Baseline characteristics of the STEP UP MRI subpopulation and STEP UP total population.

Characteristic	STEP UP MRI subpopulation			STEP UP
	Pooled semaglutide 7.2 mg/2.4 mg (*n* = 49)	Placebo (*n* = 6)	Total (*N* = 55)	Total population (*N* = 1407)
Sex, *n* (%)				
Female	39 (79.6)	4 (66.7)	43 (78.2)	1037 (73.7)
Male	10 (20.4)	2 (33.3)	12 (21.8)	370 (26.3)
Race, *n* (%)				
White	43 (87.8)	4 (66.7)	47 (85.5)	1203 (85.5)
Black or African American	6 (12.2)	2 (33.3)	8 (14.5)	121 (8.6)
Ethnicity, *n* (%)				
Hispanic or Latino	5 (10.2)	0	5 (9.1)	63 (4.5)
Not Hispanic or Latino	44 (89.8)	6 (100.0)	50 (90.9)	1344 (95.5)
Age, years	45 (12)	43 (9)	45 (12)	47 (12)
Bodyweight, kg	109.9 (19.3)	117.9 (23.1)	110.8 (19.7)	113.0 (24.1)
BMI, kg/m^2^	39.0 (5.8)	42.1 (6.9)	39.3 (5.9)	39.9 (7.1)
Waist circumference, cm	119.5 (12.9)	121.8 (14.6)	119.8 (13.0)	118.7 (15.8)
HbA_1c_, %	5.6 (0.3)	5.6 (0.2)	5.6 (0.3)	5.7 (0.3)
HbA_1c_, mmol/mol	37.7 (3.4)	38.1 (1.9)	37.8 (3.3)	38.4 (3.7)
Glycaemic status at baseline^a^, *n* (%)				
Normoglycaemia	30 (61.2)	4 (66.7)	34 (61.8)	877 (62.3)
Prediabetes	19 (38.8)	2 (33.3)	21 (38.2)	529 (37.6)
eGFR, mL/min per 1.73 m^2^ ^b^	101.44 (16.6)	88.82 (35.6)	99.98 (19.4)	95.3 (19.5)
Body composition				
Adipose body tissue volume, L	42.9 (9.6)	47.1 (10.1)	43.4 (9.7)	—
Visceral adipose tissue volume, L	5.4 (1.7)	5.1 (2.5)	5.3 (1.8)	—
Lean body tissue volume, L	23.8 (5.6)	24.5 (6.0)	23.9 (5.6)	—
Thigh skeletal muscle volume, L	10.9 (2.7)	11.7 (3.1)	11.0 (2.7)	—
*z*‐score^c^	0.16 (1.15)	0.46 (1.50)	0.31 (1.33)	—
Thigh muscle fat infiltration, %	9.44 (1.90)	9.76 (1.08)	9.47 (1.82)	—

*Note:* MRI scans were from the neck to the knees; adipose and lean body tissue volumes were measured from thoracic vertebra T9 to the knees without extrapolation to the whole body. Data are *n* (%) or mean (standard deviation) unless otherwise stated.

Abbreviations: BMI, body mass index; eGFR, estimated glomerular filtration rate; HbA_1c_, glycated haemoglobin; MRI, magnetic resonance imaging.

^a^ Evaluated by the Investigator based on all available or relevant information.

^b^ Geometric mean (coefficient of variation).

^c^ *z*‐scores are the deviation from the mean value of a matched group (sex and BMI), expressed as the number of standard deviations.

Pooled semaglutide was associated with a significantly larger reduction in mean bodyweight (19.5 kg, 17.4%) as compared with placebo (0.4 kg, 0.9%) (*p* = 0.004, *p* < 0.001) (Table 2).

**TABLE 2:** Baseline and change in MRI‐defined body composition endpoints from baseline to Week 72 in the STEP UP MRI subpopulation (treatment policy estimand).

	Pooled semaglutide 7.2 mg/2.4 mg (*n* = 49)^a^	Placebo (*n* = 6)^a^	ETD (95% CI) (pooled semaglutide vs. placebo)	*p*‐value comparison (pooled semaglutide vs. placebo)
Total bodyweight				
Baseline, mean (SD), kg	109.9 (19.3)	117.9 (23.1)	—	—
Absolute change, kg	−19.5 (−24.0 to −15.1)	−0.4 (−12.8 to 11.9)	−19.1 (−32.1 to −6.2)	*p* = 0.004
Relative change, %	−17.4 (−21.4 to −13.4)	−0.9 (−10.0 to 8.3)	−16.5 (−26.3 to −6.8)	*p* = 0.0009
Adipose body tissue volume				
Baseline, mean (SD), L	42.9 (9.6)	47.1 (10.1)	—	—
Absolute change, L	−11.0 (−13.5 to −8.6)	0.1 (−4.7 to 4.8)	−11.1 (−16.5 to −5.7)	*p* < 0.0001
Relative change, %	−25.1 (−30.6 to −19.6)	−0.9 (−9.6 to 7.8)	−24.3 (−34.7 to −13.9)	*p* < 0.0001
Visceral adipose tissue volume				
Baseline, mean (SD), L	5.4 (1.7)	5.1 (2.5)	—	—
Absolute change, L	−1.6 (−2.1 to −1.2)	−0.1 (−1.5 to 1.2)	−1.5 (−2.9 to −0.1)	*p* = 0.04
Relative change (%)	−31.3 (−39.7 to −22.8)	−6.3 (−27.9 to 15.2)	−24.9 (−48.7 to −1.1)	*p* = 0.04
Lean body tissue volume				
Baseline, mean (SD), L	23.8 (5.6)	24.5 (6.0)	—	—
Absolute change, L	−1.7 (−2.3 to −1.0)	−0.04 (−2.3 to 2.3)	−1.7 (−4.0 to 0.7)	*p* = 0.16
Relative change, %	−6.7 (−9.7 to −3.8)	0.2 (−8.9 to 9.3)	−7.0 (−16.2 to 2.2)	*p* = 0.14
Thigh skeletal muscle volume				
Baseline, mean (SD), L	10.9 (2.7)	11.7 (3.1)	—	—
Absolute change, L	−1.1 (−1.4 to −0.8)	−0.02 (−1.2 to 1.2)	−1.1 (−2.3 to 0.1)	*p* = 0.07
Relative change, %	−10.0 (−13.2 to −6.8)	−0.8 (−10.5 to 8.9)	−9.2 (−18.8 to 0.4)	*p* = 0.06
*z*‐score^b^	−0.35 (−0.53 to −0.18)	0.05 (−0.82 to 0.91)	−0.40 (−1.25 to 0.45)	*p* = 0.36
Thigh muscle fat infiltration				
Baseline, mean (SD), %	9.4 (1.9)	9.8 (1.1)	—	—
Absolute change (pp)	−0.56 (−0.74 to −0.37)	0.36 (−0.10 to 0.81)	−0.92 (−1.41 to −0.42)	*p* = 0.001
Relative change (%)	−5.78 (−7.76 to −3.79)	4.06 (−1.47 to 9.60)	−9.84 (−15.73 to −3.95)	*p* = 0.001

*Note:* MRI scans were from the neck to the knees; adipose and lean body tissue volumes were measured from thoracic vertebra T9 to the knees without extrapolation to the whole body. Data are mean (95% CI) unless otherwise indicated. Observed data are from the in‐trial period; estimated data are for the treatment policy estimand (effect regardless of trial product discontinuation, dose reduction or initiation of any other obesity therapies or surgery).

Abbreviations: CI, confidence interval; ETD, estimated treatment difference; MRI, magnetic resonance imaging; pp, percentage points; SD, standard deviation.

^a^ Participant numbers in the pooled semaglutide 7.2 mg/2.4 mg and placebo groups were: *n* = 46 and *n* = 6 for change in total bodyweight; *n* = 44 and *n* = 5 for change in thigh skeletal muscle volume *z*‐score; and *n* = 49 and *n* = 5 for all other outcomes, respectively.

^b^ *z*‐scores are the deviation from the mean value of a matched group, expressed as the number of standard deviations.

Absolute and relative changes from baseline to Week 72 in body composition endpoints (as measured from thoracic vertebra T9 to knees) are shown in Figure 2A,B, and Table 2. Relative changes in adipose and lean body tissue volumes and visceral adipose volume were also reported in the primary publication [15]. The pooled semaglutide group had mean within‐group reductions in adipose body tissue volume (11.0 L, 25.1%), visceral adipose tissue volume (1.6 L, 31.3%), lean body tissue volume (1.7 L, 6.7%) and thigh skeletal muscle volume (1.1 L, 10.0%). An absolute 0.56%‐point (relative 5.78%) reduction in thigh muscle fat infiltration was also observed. In the placebo group, absolute changes were 0.1 L, −0.1 L, −0.04 L, −0.02 L and 0.36%‐points, respectively; the differences between groups for total and visceral adipose body tissue volumes, and for thigh muscle fat infiltration, were statistically significant (*p* < 0.05) (Figure 2A,B, and Table 2). The estimated proportions of weight lost from adipose and lean body tissue mass (from thoracic vertebra T9 to the knees) were 84.4% and 15.6%, respectively, in the pooled semaglutide group (Figure 3). The mean thigh skeletal muscle volume *z*‐score was reduced in the pooled semaglutide group (0.35) and increased in the placebo group (0.05), but the difference was not statistically significant (Table 2). A sensitivity analysis of the semaglutide 7.2 mg group versus the placebo group showed similar results to the pooled semaglutide group versus placebo for body composition parameters (Supporting Information, p. 11, Figure S2). Descriptive mean changes for the semaglutide 2.4 mg group showed similar but smaller changes from baseline to Week 72 compared with the semaglutide 7.2 mg group (Figure S2).

**FIGURE 2:**
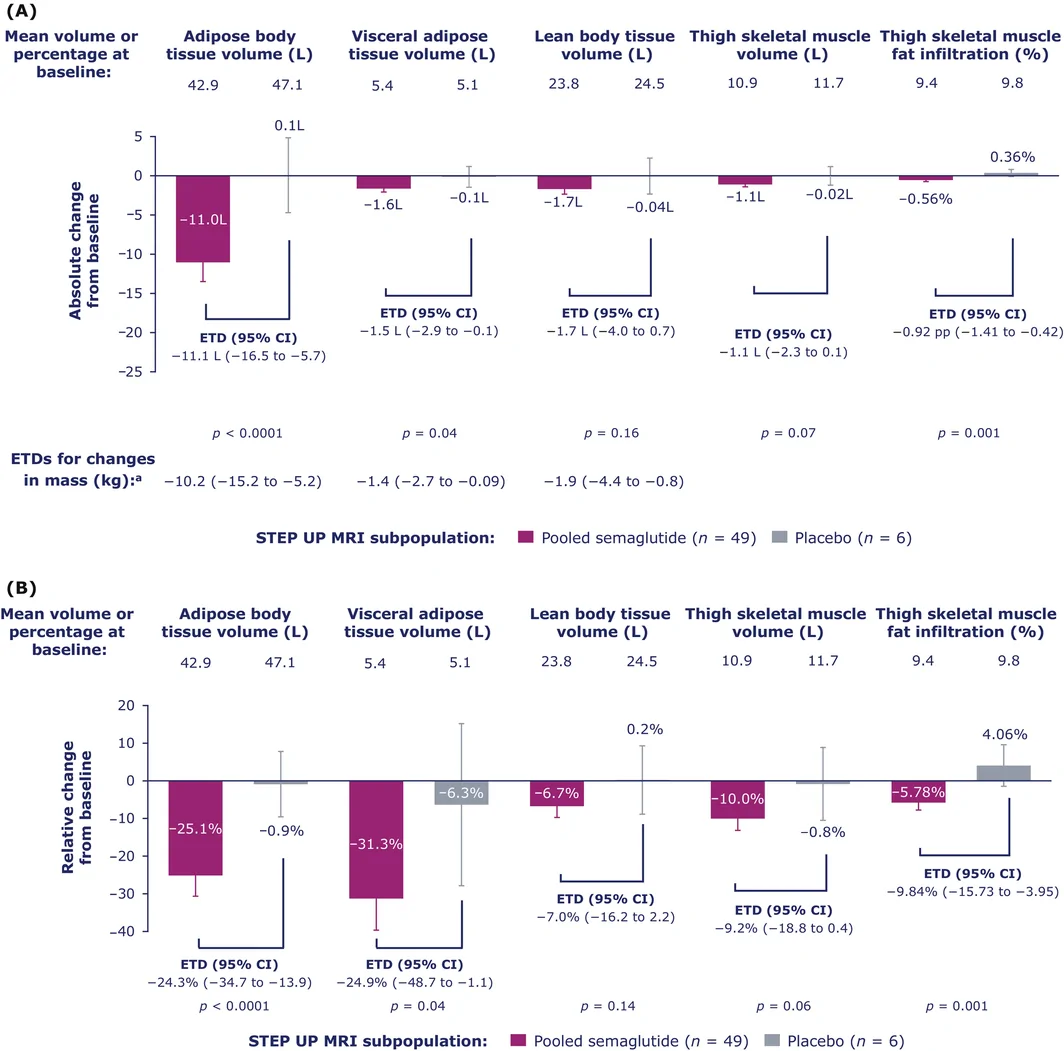
Absolute (A) and relative (B) changes in the tissue depot volumes from baseline to Week 72 with pooled semaglutide and placebo. *Note:* MRI scans were from the neck to the knees; adipose and lean body tissue volumes were measured from thoracic vertebra T9 to the knees without extrapolation to the whole body. Data are estimated using the treatment policy estimand (effect regardless of trial product discontinuation, dose reduction, or initiation of any other obesity therapies or surgery). Error bars are 95% CIs. CI, confidence interval; ETD, estimated treatment difference; MRI, magnetic resonance imaging; pp, percentage point. ^a^ETDs in adipose and lean body tissue volumes were converted to ETDs in mass by multiplying the former by the corresponding tissue densities of 0.92 (adipose tissue) or 1.1 (lean tissue) [21].

**FIGURE 3:**
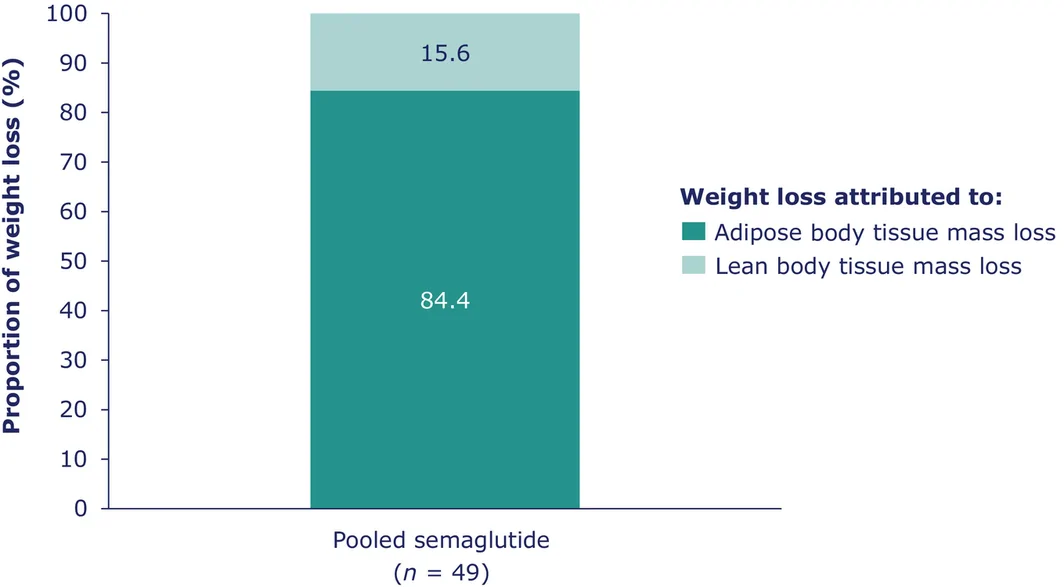
Estimated proportions of weight loss attributable to adipose or lean tissue mass loss at Week 72 in the STEP UP MRI subpopulation. *Note:* Adipose and lean body tissue volumes were measured from thoracic vertebra T9 to the knees without extrapolation to the whole body. Data estimated using the treatment policy estimand (effect regardless of trial product discontinuation, dose reduction, or initiation of any other obesity therapies or surgery). The total weight loss used to calculate proportions correspond to the sum of the adipose and lean tissue mass. Adipose tissue mass loss was calculated using −10.1/(−10.1 kg + −1.9 kg)*100 = 84%. Lean tissue mass loss was calculated using −1.9/(−10.1 kg + −1.9 kg)*100 = 16%. MRI, magnetic resonance imaging.

In terms of physical function, sit‐to‐stand test data were available for 51 (92.7%; pooled semaglutide, *n* = 46; placebo, *n* = 5) of 55 participants. The mean (SD) number of repetitions was similar in the pooled semaglutide and placebo groups, both at baseline (13 [4] and 13 [2] per 30 s) and Week 72 (15 [4] and 15 [1] per 30 s), respectively (Supporting Information, p. 13, Figure S3). A sensitivity analysis showed similar results for semaglutide 7.2 and 2.4 mg alone versus placebo, with a mean (SD) number of repetitions at Week 72 of 14 (4) and 16 (5) versus 15 (1), respectively.

Adverse events occurred in 95.3% of participants in the semaglutide 7.2 mg group, 83.3% of those in the semaglutide 2.4 mg group and 66.7% of those in the placebo group (Supporting Information, p. 9, Table S2). Gastrointestinal‐related adverse events occurred in 86.0%, 50.0% and 33.3% of participants in the semaglutide 7.2 mg, semaglutide 2.4 mg and placebo groups, respectively. Musculoskeletal and connective tissue disorder adverse events occurred in 7.0% of participants in the semaglutide 7.2 mg group, with none occurring in the semaglutide 2.4 mg and placebo groups.

## 4 Discussion

In this prespecified analysis of supportive secondary and exploratory endpoints of the STEP UP trial in people with obesity, participants treated with semaglutide (7.2 mg/2.4 mg) had significant reductions at Week 72, compared with the placebo group, in bodyweight (19.1 kg; 16.5%), adipose body tissue volume (11.1 L; 24.3%), visceral adipose tissue volume (1.5 L; 24.9%) and thigh muscle fat infiltration (0.92%‐points; 10%). Reductions were observed in lean body tissue volume (1.7 L; 7.0%) and thigh skeletal muscle volume (1.1 L; 9.2%), while the number of sit‐to‐stand repetitions was preserved. Together, these results indicate that treatment with semaglutide (7.2 mg/2.4 mg) as an adjunct to a reduced‐calorie diet and increased physical activity was associated with clinically important reductions in adipose tissue volume, concurrent reductions in thigh skeletal muscle volume and fat infiltration, and no deterioration in physical function.

Our findings provide insights on muscle‐related changes associated with bodyweight reduction with semaglutide as an adjunct to lifestyle intervention, giving additional nuance to previously reported concerns regarding skeletal muscle mass loss in GLP‐1RA trials (as measured by DXA) [6, 22]. Loss of muscle mass is an expected outcome of intentional bodyweight reduction, as the physical stimulation on skeletal muscle to perform activities of daily living decreases with lower bodyweight [3, 4]. In line with this, caloric restriction with subsequent weight loss is associated with loss of total body and appendicular lean mass, while concomitant exercise and/or sufficient protein intake may attenuate this effect [3]. Participants in the STEP UP trial were encouraged to increase their physical activity to at least 150 min per week, for example, walking or using the stairs, but dietary protein intake was not specifically assessed. As these parameters were not directly assessed, and when also considering the relatively small cohort sizes, the impact of individual changes in behavioural and physical activities could have influenced changes in body composition and physical function. However, a recent study demonstrated that individuals with obesity treated with semaglutide had improvements in body composition without a negative impact on strength (as measured by isometric thigh muscle strength and hand grip strength) [23]. In the present analysis and the primary STEP UP trial, participants treated with semaglutide had similar baseline and end‐of‐trial sit‐to‐stand repetitions, indicating a preservation of physical function despite substantial bodyweight reductions.

A clear consensus on what constitutes an acceptable degree of skeletal muscle mass loss following bodyweight reduction is missing from the current literature. Many studies have systematically evaluated specified outcomes related to body composition, with differences in methodologies, study populations and reporting standards, but few studies have assessed skeletal muscle quantity, quality and physical function. To the best of our knowledge, only two randomised clinical trials have reported changes in MRI‐based measures of thigh skeletal muscle volume and muscle fat infiltration after treatment with GLP‐1 (liraglutide) or dual GLP‐1/GIP (tirzepatide) RAs, which are discussed below. Further complexity is added by the fact that change in total lean mass (or total fat‐free mass, both traditionally assessed by DXA) does not only measure changes in skeletal muscle mass as it also includes organs, water in adipose tissue, connective tissue and fluids, whereas MRI provides detailed body composition assessment through precise anatomical separation and quantification of various tissues (e.g., muscle, adipose) and organ volumes. While DXA remains the most accessible image‐based assessment of body composition (at the molecular level) due to lower cost, MRI offers greater accuracy and precision [24, 25]. Although studies often show strong and significant correlations between measurements from DXA and MRI (or computed tomography), inconsistencies between the techniques are commonly reported, including overestimation of subcutaneous adipose tissue and underestimation of visceral adipose tissue with DXA [26].

Studies utilising whole‐body skeletal muscle assessment are scarce due to the limitations of the imaging techniques such as radiation risk and costly time‐intensive protocols. The MRI‐based body composition assessment utilised in STEP UP was derived from a standardised image acquisition protocol covering neck to knees, combined with an analysis workflow purposefully designed for rapid, large‐scale application and comparable results across studies, scanner manufacturers and field strengths. This allowed for standardised implementation across all sites; however, due to the lack of appendicular scanning, changes in lean body tissue volume in this study may not necessarily correspond to total lean body tissue volume.

Although accurate measurement of skeletal muscle mass in clinical settings is more challenging, the integration of MRI substudies into trials of obesity medications is increasingly used in lieu of DXA to characterise changes in skeletal muscle volume and muscle fat infiltration after weight‐loss interventions, with more recent clinical trials evaluating additional methodologies (i.e., computed and positron emission tomography scans and D3 creatinine). These outcomes are important for clinical practice as sarcopenia (the loss of skeletal muscle mass and strength) and myosteatosis (the infiltration of fat into the muscle) have been consistently linked to physical disability, poor quality of life and increased mortality risk [18]. Poor muscle quality is inherently underdiagnosed in people with obesity, mostly as a consequence of heterogeneous assessment, warranting standardisation in both the clinic and clinical trial setting [13]. Historically, myosteatosis has been quantified through different approaches, and the terminology used in literature is inconsistent [27]. Muscle fat infiltration, as measured in this study, captures what is commonly called *intra*muscular fat, that is, the visible storage of lipids in adipocytes located between the muscle fibres, while excluding lipids in adipocytes located between muscle groups (literally *inter*muscular fat). This does not include separation of intramyocellular lipids (a smaller group of lipids stored within the muscle cells themselves), which requires more specialised imaging (e.g., magnetic resonance spectroscopy). Preliminary data in weight‐stable individuals without metabolic disease show that muscle fat infiltration increased by mean (SD) 0.11 (0.17)%‐points during 1 year of ageing [28]. This suggests that the *decrease* seen with semaglutide in STEP UP was 3–4 times greater than the *increase* seen with ageing. Further, published results on all‐cause mortality in a cohort of ~40 000 participants (mean age 64 years; BMI 26.4 kg/m^2^) indicate that a 1%‐point difference in muscle fat infiltration was associated with an 11% increased risk of death (when adjusted for sex, age, BMI, muscle volume *z*‐score and hand grip strength) [18].

This prespecified analysis of changes in body composition in a subgroup undergoing MRI in the STEP UP trial used a similar AMRA methodology to two previously published clinical trials. In a *post hoc* analysis of SURPASS‐3, participants with type 2 diabetes and elevated liver fat content treated with pooled tirzepatide 15 mg had mean reductions in bodyweight of 9.6 kg, thigh skeletal muscle volume of 0.64 L, muscle volume *z*‐score of 0.22 and muscle fat infiltration of 0.36%‐points [29]. Further, in an analysis of another trial, participants with overweight or obesity without type 2 diabetes treated with liraglutide 3 mg had a reduction in thigh muscle fat infiltration of 0.26%‐points, which correlated with bodyweight reduction (6.6%), and a reduction in the proportion of participants with adverse muscle composition (i.e., low muscle volume *z*‐score and high muscle fat infiltration), compared with placebo [9, 30]. Although these studies cannot be directly compared due to differences in treatments, populations, trial durations and magnitude of bodyweight reduction, they contribute to the emerging evidence regarding the potential beneficial effects of GLP‐1 and GLP‐1/GIP RAs on muscle quality (reduced muscle fat infiltration). A summary of key muscle outcomes from studies including tirzepatide and liraglutide and from our study is shown in Table S1 (Supporting Information, p. 8).

Also, treatment with semaglutide was not associated with a reduced number of sit‐to‐stand repetitions, neither in absolute numbers nor in comparison with placebo. These data are similar to what was observed in the primary trial, with both semaglutide groups and the placebo group having small increases in the number of sit‐to‐stand repetitions [15]. Previous studies have demonstrated that among healthy individuals, those aged 20–59 years had sit‐to‐stand repetition values of 24 (males) and 23 (females), whereas those aged ≥ 60 years had values of 18 and 16, respectively [31]. In a small‐scale study of 32 adults with obesity (81% female, mean age 50 years and BMI 44 kg/m^2^), the mean number of repetitions in a 30‐s sit‐to‐stand test was 16–17 [32]. These data indicate that the baseline level of sit‐to‐stand repetitions in the present analysis was below that seen previously. Prior evidence has shown that a change of at least two repetitions in the 30‐s sit‐to‐stand test (in those with hip osteoarthritis) corresponded to a minimally clinically important difference [33]. The change in sit‐to‐stand scores in the present analysis indicated no statistically significant change in scores. These data warrant further exploration, as to date only a paucity of studies have assessed the combination of muscle quantity, quality and function in people with obesity. Improvement in muscle quality may be a key therapeutic strategy in the clinical setting [34].

Strengths of this study include the MRI‐based muscle metrics, offering high precision and reproducibility in assessment, combined with physical testing, providing indicative results on muscle quality and physical function. Limitations include that, although prespecified, this study was a secondary analysis with a small sample size, particularly in the placebo group and has limited statistical power. This restricts the ability to make robust comparisons between groups, and therefore, the data should be treated as exploratory and hypothesis generating. Additionally, the study sample may not be representative of the general population with obesity, as several metabolic and chronic diseases were excluded due to the parent study's inclusion/exclusion criteria and most participants were female (78.2% [43 of 55]), White (85.5% [47 of 55]), relatively young (mean [SD] age 47 [12] years) and had a bodyweight < 149 kg. Future studies should investigate the effect of semaglutide on muscle quantity, quality and physical function in more vulnerable subgroups of obesity with an elevated risk of sarcopenia, such as the elderly. In addition, the specific bodily region used to measure adipose and lean body tissue volumes (thoracic vertebra T9 to the knees) has a higher adipose:lean volume ratio compared with other regions that were not measured (calves, arms, shoulders and head). Thus, the reported numbers likely underestimate total lean body mass loss. It is unlikely that this fully explains the large magnitude of difference in lean body mass loss compared with what was reported for STEP 1 [10]. However, without harmonisation in study populations, designs and measurement techniques, it is difficult to conclude what the true lean body mass loss associated with semaglutide was. As the use of MRI to assess body composition is increasing across interventional clinical trials, the results from this study can provide reference data for future studies. The sit‐to‐stand test was used as a marker of physical function; however, this technique may have had confounders introduced due to the participant population potentially adapting to the test following the initial baseline assessment. Additionally, increases in sit‐to‐stand repetitions may be more influenced by bodyweight loss than preservation of skeletal muscle function. Further, patient‐reported outcomes that included self‐reported physical function domains were not included in the present analysis. Lifestyle and behavioural changes, such as dietary protein intake and physical activity, which may have provided further insights into muscle mass preservation, were not assessed in this study. Future studies should include more specific assessments of physical function and strength that are relevant to the individual (e.g., sit‐to‐stand, hand grip strength and 400‐m walk test) and lifestyle and behavioural changes that may affect muscle mass preservation during weight loss, alongside measurements of metabolic muscle health. However, it should be noted that the aims of the present analysis were not to assess improvements in physical function but rather to assess whether physical function was maintained following semaglutide‐driven weight loss.

## 5 Conclusion

In summary, this secondary analysis of MRI data from the STEP UP trial showed that participants treated with semaglutide as an adjunct to lifestyle intervention lost substantial body and visceral adipose tissue volumes. In addition, we report decreases in both thigh skeletal muscle volume and thigh muscle fat infiltration with preserved physical function as indicated by the sit‐to‐stand test. Larger studies with adequately powered cohorts directly measuring skeletal muscle mass/volume, muscle fat infiltration and skeletal muscle function to investigate the impact of weight loss on these outcomes are needed.

## Author Contributions

J.H., W.T.G., I.L., J.R. and S.W. were involved in the conduct of the trial and contributed to data collection. S.B. performed the data analysis, and directly accessed and verified the data. All authors had full access to all the data in the trial and had final responsibility for the decision to submit the manuscript for publication. All authors contributed to data interpretation and writing of the manuscript (assisted by a medical writer paid for by the funder), and vouch for data accuracy and fidelity to the protocol. All authors approved the final version of the manuscript.

## Funding

This work was supported by Novo Nordisk A/S.

## Conflicts of Interest

Jøran Hjelmesæth has received fees or honoraria for academic presentations from Novo Nordisk A/S, Eli Lilly & Co, VIVUS, and AstraZeneca; and has received fees or honoraria to participate on advisory boards for Novo Nordisk, Eli Lilly & Co, and VIVUS, with all payments made to his institution since 2024. Suhas Bhat is an employee of Novo Nordisk. W. Timothy Garvey has received grants as a principal investigator from Novo Nordisk, Eli Lilly & Co, Pfizer, Epitomee, Neurovalens, Zealand, Carmot/Roche, Terns Pharmaceuticals, Viking Therapeutics, and Pfizer, and for research from the UAB Diabetes Research Center (DK079626) funded by the US National Institutes of Health and National Institute of Diabetes and Digestive and Kidney Diseases, with all payments made to his institution; has received consulting fees as an advisory board member from Boehringer Ingelheim, Eli Lilly & Co, Novo Nordisk, Pfizer, Fractyl Health, Alnylam Pharmaceuticals, Inogen, Zealand, Allurion, Carmot/Roche, Terns Pharmaceuticals, Neurocrine, KEROS Therapeutics, Gan & Lee, NodThera, and Regeneron; has received fees to participate on a data safety monitoring board or advisory board for Boehringer Ingelheim and Eli Lilly & Co; and is an unpaid member of *The Lancet Diabetes & Endocrinology* Commission on definition and diagnostic criteria of clinical obesity, American Association of Clinical Endocrinology (AACE) update of obesity treatment algorithm, American Diabetes Association (ADA) nutrition recommendations update, and ADA Standards of Care for Obesity. Kristoffer Jensen Kolnes is a full‐time employee of and holds shares in Novo Nordisk. Jennifer Linge is an employee and shareholder of AMRA Medical AB, and a consultant for Eli Lilly & Co. Ildiko Lingvay has received grants from Boehringer Ingelheim, Dexcom, Eli Lilly & Co, Novo Nordisk, Pfizer, and Roche, with all payments made to her institution; has received payment or honoraria from Aardvarak Therapeutics, AbbVie, Adarx, Altasciences, Alveus Therapeutics, Amgen, Antag Therapeutics, Arrowhead Pharmaceuticals, AstraZeneca, Baim Institute, Bain Capital, Bayer, Betagenon AB, Bioio Inc., Biomea, Boehringer Ingelheim, Boston Scientific, Carmot, Corxel, Cytoki Pharma, Eli Lilly & Co, Genentech, Janssen/J&J, Juvena, Kailera Therapeutics, Keros Therapeutic Inc., Mediflix, Metsera, Neurocrine, Novo Nordisk, Penguin Bio, Pfizer, Regeneron, Response Pharmaceutical, Roche, Sanofi, Shionogi, Skye Bioscience, Source Bio, Sparrow, Structure Therapeutics, SUMMIT, Tenvie, Terns Pharma, Verdiva Bio, Viking Therapeutics, and Zealand Pharma. Niklas Kahr Rasmussen is a full‐time employee of, and holds shares in, Novo Nordisk. Julio Rosenstock has received research grants from Amgen, Applied Therapeutics, AstraZeneca, Biomea Fusion, Boehringer Ingelheim, Carmot, Corcept, Eli Lilly & Co, Hanmi, Merck, Novartis, Novo Nordisk, Oramed, Regeneron, Pfizer, Sanofi, Structure Therapeutics, and Terns Pharmaceuticals with all payments to Velocity Clinical Research; has served on scientific advisory boards for and received honoraria or consulting fees from Amgen, Applied Therapeutics, Biomea Fusion, Eli Lilly & Co, Eccogene, Hanmi, Novo Nordisk, Regeneron, Regor, Roche, Sanofi, Structure Therapeutics, and Terns Pharmaceuticals; and has received honoraria for lectures from Eli Lilly & Co, Novo Nordisk, and Sanofi. Sean Wharton has received payment or honoraria from AbbVie, Amgen, AstraZeneca, Bausch Health Canada, Boehringer Ingelheim, Eli Lilly, i2o Therapeutics, Merck, Metsera, NGX, Novo Nordisk, and Regeneron for academic talks to colleagues; has been provided with medical writing support from AstraZeneca, Boehringer Ingelheim, Bausch Health Canada, Eli Lilly & Co, and Novo Nordisk; and has unpaid leadership roles in The Obesity Society and Obesity Canada.

## Supporting information


**Data S1:** Supporting Information.
**Table S1:** Changes in bodyweight, thigh skeletal muscle volume and muscle fat infiltration in different MRI study populations.
**Table S2:** Adverse events in the STEP UP MRI subpopulation.
**Figure S1:** Example MRI scan (neck to knees) showing segmentations of total adipose tissue, visceral adipose tissue, lean tissue and thigh skeletal muscle volumes in one STEP UP trial participant at the baseline and follow‐up examinations.
**Figure S2:** Sensitivity analysis of absolute (A) and relative (B) changes in the tissue depot volumes from baseline to Week 72 with semaglutide 7.2 mg, semaglutide 2.4 mg and placebo.
**Figure S3:** Sit‐to‐stand test repetitions at baseline and Week 72.

## Data Availability

An authorised researcher can request access to clinical study data by submitting a research proposal for review and approval by Novo Nordisk and an internal independent review panel. Requests are usually considered after the research is finished and the main results have been published. If the research supports a regulatory application, requests will be considered after the product and its intended use are approved in both the EU and the USA. Participants' clinical data will be anonymised, following an approved internal process, before data are shared with external third parties. For details on how to request access to clinical data, visit novonordisk‐trials.com.
