# Supplementary material for: Effect of Semaglutide on Thigh Skeletal Muscle Volume, Fat Infiltration and Physical Function in People With Obesity: Secondary Analysis of STEP UP
Source: Diabetes Obes Metab. 2026 Sep 20;28(12):11653–63. doi: 10.1111/dom.71323 (PMC13731004; doi:10.1111/dom.71323)
Supplement: Supplementary file 1 [file DOM-28-11653-s001.docx]

**Supporting information**

**Effect of Semaglutide on Thigh Skeletal Muscle Volume, Fat Infiltration and Physical Function in People With Obesity:
Secondary Analysis of STEP UP**

Jøran Hjelmesæth, MD, PhD^1,2^, Suhas Bhat, PhD^3^, W. Timothy Garvey, MD^4^, Kristoffer Jensen Kolnes, MD, PhD^5^, Jennifer Linge, PhD^6,7^, Ildiko Lingvay, MD^8^, Niklas Kahr Rasmussen, MD^5^, Julio Rosenstock, MD^9^, Sean Wharton, MD^10^

^1^Department of Endocrinology, Obesity and Nutrition, Vestfold Hospital Trust, Tønsberg, Norway

^2^Department of Endocrinology, Morbid Obesity and Preventive Medicine, Institute of Clinical Medicine, University of Oslo, Oslo, Norway

^3^Novo Nordisk Global Business Services, Bangalore, India

^4^University of Alabama at Birmingham, Birmingham, AL, USA

^5^Novo Nordisk A/S, Bagsværd, Denmark

^6^AMRA Medical AB, Linköping, Sweden

^7^Department of Health, Medicine and Caring Sciences, Linköping University, Linköping, Sweden

^8^Department of Internal Medicine/Endocrinology and Peter O’Donnell Jr. School of Public Health, University of Texas Southwestern Medical Center, Dallas, TX, USA

^9^University of Texas Southwestern Medical Center, Dallas, TX, USA

^10^University of Toronto, Toronto, ON, Canada

**Correspondence:** Professor Jøran Hjelmesæth, MD

**Address:** Department of Endocrinology, Obesity and Nutrition

EASO Collaborating Adult and Paediatric Centre for Obesity Management

Vestfold Hospital Trust

Boks 2168, 3103 Tønsberg, Norway

**Tel:** +47 40 21 73 49

**Email:** [joran.hjelmeseth@siv.no](mailto:joran.hjelmeseth@siv.no)

Contents

[Contents	2](#_Toc239704281)

[Additional Methodology	3](#_Toc239704282)

[Randomisation and Masking	3](#_Toc239704283)

[AMRA Methodology	3](#_Toc239704284)

[Statistical Methods	6](#_Toc239704285)

[TABLE S1 Changes in bodyweight, thigh skeletal muscle volume and muscle fat infiltration in different MRI study populations.	8](#_Toc239704286)

[TABLE S2 Adverse events in the STEP UP MRI subpopulation.	9](#_Toc239704287)

[FIGURE S1 Example MRI scan (neck to knees) showing segmentations of total adipose tissue, visceral adipose tissue, lean tissue and thigh skeletal muscle volumes in one STEP UP trial participant at the baseline and follow-up examinations.	10](#_Toc239704288)

[FIGURE S2 Sensitivity analysis of absolute (A) and relative (B) changes in the tissue depot volumes from baseline to Week 72 with semaglutide 7.2 mg, semaglutide 2.4 mg and placebo.	11](#_Toc239704289)

[FIGURE S3 Sit-to-stand test repetitions at baseline and Week 72.	13](#_Toc239704290)

[References	14](#_Toc239704291)

## Additional Methodology

### Randomisation and Masking

Participants were advised not to initiate any other obesity therapies. Visually identical injections were used to maintain masking of participants and site staff. Participants followed a fixed-dose escalation regimen, starting with semaglutide 0.25 mg and increasing every 4 weeks to 2.4 mg at Week 16. Participants in the 2.4 mg group remained on 2.4 mg from Week 16, and at Week 20, participants in the semaglutide 7.2 mg group directly escalated to this dose without additional titration. Participants were encouraged to remain in the trial regardless of whether they had to discontinue the trial product permanently.

Regarding substudy enrolment, as enrolment depended on both the participant and the investigator, this can be considered convenience sampling. After the inclusion criteria were checked, participants decided whether to consent to the magnetic resonance imaging (MRI) scans; if they were willing, they would proceed. Therefore, the MRI scans depended on the participant consent process. Further, as the trial was blinded, inclusion was not based on which treatment arm the participant was randomised to.

### AMRA Methodology

The MRI data were acquired with standardised imaging parameters and scanning procedures as described previously.^1^ Visceral adipose tissue, skeletal muscle volume, and muscle fat infiltration measurements have been extensively validated (including accuracy, repeatability, and reproducibility studies) in the literature, illustrating the reliability of the MRI quantification.^1-8^ Adipose and lean body tissue volumes (measured from thoracic vertebra T9 to the knees), visceral adipose tissue volume, and thigh muscle composition (skeletal muscle volume, muscle fat infiltration, and muscle volume *z*-score, i.e., to compare an individual’s thigh skeletal muscle volume with those of a matched group) were measured. Thigh muscle measured included the following muscles: quadriceps femoris, sartorius, tensor fasciae latae, gluteus, iliacus, adductor, and hamstring.^9^ The MRI-based body composition assessment utilised in STEP UP was derived from a standardised image acquisition protocol covering neck to knees, combined with an analysis workflow purposefully designed for rapid, large-scale application and comparable results across studies, scanner manufacturers, and field strengths. Additionally, the calves were excluded to reduce scan time and ensure consistency across the range of scanners (some of which have limited table travel length) used within the study. The arms were also excluded to avoid the need for repositioning the participant, practically simplifying image acquisition and analysis, lowering the risk of introducing variability, and significantly shortening the total scan time.

On all scanners, the body-MRI protocol was based on fat-water separated 3D complex-based two-point chemical shift encoded MRI, also known as 2-point Dixon water-fat separation imaging, with neck to the knee scan coverage and adjacent slab overlap of 2–4 cm. All neck to the knee images were T1-weighted with 10° flip angle and repetition time set to shortest possible. Fat, water, in-phase, and opposite-phase images were acquired where fat and water images were reconstructed using the on-scanner software for all acquisitions. All scans were performed in supine position using the integrated body coils. Body composition analysis of the collected images was performed with AMRA Researcher (AMRA Medical AB, Linköping, Sweden) in four steps:^10^ calibration using fat signal interpolation from pure adipose tissue as a reference (fat-referenced MRI); image segmentation using an automated atlas-based approach; quality control by two independent, trained operators at AMRA; and quantification of fat and muscle metrics within the segmented regions using dedicated algorithms.

The following measurements were extracted: adipose body tissue volume, lean body tissue volume, visceral adipose tissue volume, thigh skeletal muscle volume (total fat-free thigh muscle volume), and thigh muscle fat infiltration (fat fraction within the muscle volume region). In brief, the analysis consists of calibration using fat-referenced MRI to obtain quantitative fat-based concentration images, merging to obtain the full coverage in one image volume, segmentation using an automated atlas-based approach, manual control of image and segmentation quality by two operators, and extraction of the measurements using dedicated algorithms. The analysis has been described in more detail previously.^1^ The total adipose tissue and lean body tissue volumes were measured from the top of T9 vertebrae to the lower end of thigh skeletal muscle (see **Figure S1**).

The adipose body tissue volume was calculated by integrating the quantitative fat image over the region.^10^ Similarly, total lean tissue was calculated by subtracting the total fat volume from the total soft tissue volume in the same region.^11^ These measurements have previously been described and validated against dual-energy x-ray absorptiometry DXA-based fat and lean mass.^10^ An example of total adipose tissue and total lean tissue assessment in one participant is shown in **Figure S1**. The visceral adipose tissue volume was calculated by integrating the quantitative fat image over the adipose tissue in the abdominal cavity,^2^ the thigh skeletal muscle volume was calculated as the total volume of voxels with a fat fraction below 50% within the thigh masks,^3^ and the muscle fat infiltration as the average proton density fat fraction in the thigh skeletal muscle volume region.^4^ The visceral adipose tissue volume, thigh skeletal muscle volume, and thigh muscle fat infiltration measurements have been extensively validated in the literature.^1-8^

Muscle volume *z*-scores (expressed as a number of standard deviation) were calculated based on thigh skeletal muscle volume to provide sex-, height-, weight-, and body mass index (BMI)-invariant measures of muscle volume for each participant, expressed as the deviation from the expected (mean) value of a sex- and BMI-matched virtual control group,^9^ with a score of zero indicating a muscle volume as expected, and negative or positive scores indicating lower or higher muscle volumes than expected, respectively.^12^

### Statistical Methods

Endpoints were analysed in the MRI analysis set, which included all randomly assigned participants in the subpopulation of the full analysis set that had a valid MRI scan performed at baseline.

Two observation periods were defined: the in-trial period included all on- or off-treatment data points collected during the uninterrupted period from the date of randomisation until the last trial visit, regardless of discontinuation, dose reduction, or initiation of other obesity therapies (including medications or bariatric surgery); the on-treatment period included all on-treatment data points collected during the time from the date of first trial-product administration to the date of last trial-product administration, including an ascertainment window of 14 days (efficacy) and 63 data days (adverse events). The latter excluded any off-treatment time intervals of at least two consecutive missed doses, and regardless of discontinuation, dose reduction, or initiation of other obesity therapies.

Assessments from the in-trial period were analysed according to the treatment policy estimand, which assessed treatment effect among all randomly assigned participants, regardless of trial-product discontinuation, dose reduction, or initiation of any other obesity therapies.

MRI-defined body composition changes were assessed as mean absolute changes (litres, L) and mean relative (%) changes from baseline to Week 72. The following MRI outcomes were measured, as previously described:^10^ total adipose tissue volume (L), defined as the total amount of adipose tissue in the region between T9 and the lower end of the thigh muscle; lean body tissue volume (L), defined as the total amount of lean tissue in the region between T9 and the lower end of the thigh muscle (soft tissue volume-total fat volume); visceral adipose tissue volume (L), defined as the amount of adipose tissue in the abdominal cavity;^2^ thigh skeletal muscle volume (muscle volume; L), defined as the total fat-free thigh muscle volume;^3^ and thigh muscle fat infiltration (%), defined as the fat fraction within the muscle volume of the thigh region.^4^ MRI endpoints were not included in the hierarchical testing.

The proportions of weight loss attributable to adipose or lean mass loss were estimated as:

$$Proportion of weight lost from adipose tissue mass loss \left[ \% \right]= \left\{ \left( change in adipose tissue volume \left[ L \right]\times0.92 \right)\div\left[ \left( change in adipose tissue volume \left[ L \right]\times0.92 \right)+ \left( change in lean tissue volume \left[ L \right]\times1.1 \right) \right] \right\}$$

**TABLE S1 Changes in bodyweight, thigh skeletal muscle volume and muscle fat infiltration in different MRI study populations.**

| **Assigned treatment** | **Pooled semaglutide  7.2/2.4 mg or placebo** | **Pooled tirzepatide  5/10/15 mg or insulin degludec** | **Liraglutide  3 mg or placebo** |
| --- | --- | --- | --- |
| **Study name (year)** | STEP UP MRI | SURPASS-3 MRI^13^ | MRI secondary analysis^14,15^ |
| **Trial population** | BMI ≥30 kg/m² | BMI ≥25 kg/m² and T2D | BMI ≥30 kg/m² or ≥27 kg/m²  and metabolic syndrome |
| **Treatment duration** | 72 | 52 | 40 |
| **MRI subpopulation (n for the treatment policy estimand/ITT)** | 55 | 246 | 128 |
| **Bodyweight, kg** | –19.5 (–24.0 to –15.1) | –9.6 (–10.6 to –8.5) | –6.6 |
| **Thigh skeletal muscle volume, L** | –1.1 (–1.4 to –0.8) | –0.64 (–0.74 to –0.54) | –0.35 (0.35)^a^ |
| ***z*-score^b^** | –0.35 (–0.53 to –0.18) | –0.22 (–0.29 to –0.15) | –0.11 (0.31)^a,c^ |
| **Thigh muscle fat infiltration, pp** | –0.56 (–0.74 to –0.37) | –0.36 (–0.48 to –0.25) | –0.26 (0.43) |

*Note:* Absolute changes are shown as mean (95% confidence interval) or mean (standard deviation) within-group differences for pooled semaglutide 7.2/2.4 mg, pooled tirzepatide 5/10/15 mg and liraglutide 3 mg.
Abbreviations: BMI, body mass index; ITT, intention to treat; MRI, magnetic resonance imaging; pp, percentage point; T2D, type 2 diabetes.
^a^Mean difference adjusted for baseline muscle volume/muscle volume *z*-score.
^b^*z*-scores are the deviation from the mean value of a matched group, expressed as the number of standard deviations.
^c^Change in thigh skeletal muscle volume is the least-square means difference adjusted for baseline muscle volume score.

**TABLE S2 Adverse events in the STEP UP MRI subpopulation.**

|  | **Semaglutide 7.2 mg (*n* = 43)** | | | | **Semaglutide 2.4 mg (*n* = 6)** | | | | **Placebo (*n* = 6)** | | | |
| --- | --- | --- | --- | --- | --- | --- | --- | --- | --- | --- | --- | --- |
|  | N | % | E | R | N | % | E | R | N | % | E | R |
| **Any adverse event** | 41 | 95.3 | 196 | 330.8 | 5 | 83.3 | 25 | 268.6 | 4 | 66.7 | 12 | 147.6 |
| **Serious adverse events** | 1 | 2.3 | 1 | 1.7 | 0 | - | - | - | 0 | - | - | - |
| **Adverse events leading to trial-product discontinuation** | 3 | 7.0 | 3 | 5.1 | 0 | - | - | - | 0 | - | - | - |
| **Fatal events** | 0 | - | - | - | 0 | - | - | - | 0 | - | - | - |
| **GI adverse events** | 37 | 86.0 | 99 | 167.1 | 3 | 50.0 | 12 | 128.9 | 2 | 33.3 | 3 | 36.9 |
| **Musculoskeletal and connective tissue disorder adverse events** | 3 | 7.0 | 4 | 6.8 | 0 | - | - | - | 0 | - | - | - |
| **Muscle spasms** | 2 | 4.7 | 2 | 3.4 | 0 | - | - | - | 0 | - | - | - |
| **Musculoskeletal pain** | 1 | 2.3 | 1 | 1.7 | 0 | - | - | - | 0 | - | - | - |
| **Tendonitis** | 1 | 2.3 | 1 | 1.7 | 0 | - | - | - | 0 | - | - | - |

*Note:* Adverse events from the on-treatment period.

Abbreviations: E, number of events; GI, gastrointestinal; MRI, magnetic resonance imaging; R, event rate per 100 years.

**FIGURE S1 Example MRI scan (neck to knees) showing segmentations of total adipose tissue, visceral adipose tissue, lean tissue and thigh skeletal muscle volumes in one STEP UP trial participant at the baseline and follow-up examinations.**

*Note:* Example of MRI scans (neck to knees) showing segmentations of total adipose tissue (TAT), visceral adipose tissue (VAT), total lean tissue (TLT) and thigh skeletal muscle (MV) volumes, and percentage of muscle fat infiltration in one STEP UP trial participant at the baseline and follow-up examinations. The segmentation is coloured in blue and overlaid on the fat and water images.
Abbreviations: BMI, body mass index; MRI, magnetic resonance imaging.

**FIGURE S2 Sensitivity analysis of absolute (A) and relative (B) changes in the tissue depot volumes from baseline to Week 72 with semaglutide 7.2 mg, semaglutide 2.4 mg and placebo.**

**A**

**B**

*Note:* MRI scans were from the neck to the knees; adipose and lean body tissue volumes were measured from thoracic vertebra T9 to the knees without extrapolation to the whole body. Data are estimated using the treatment policy estimand (effect regardless of trial product discontinuation, dose reduction, or initiation of any other obesity therapies or surgery). Sensitivity analysis was performed between the semaglutide 7.2 mg and placebo group only, with semaglutide 2.4 mg data included for completeness. Error bars are 95% CIs.
Abbreviations: CI, confidence interval; ETD, estimated treatment difference; MRI, magnetic resonance imaging; pp, percentage point.
^a^ETDs in adipose and lean tissue volumes were converted to ETDs in mass by multiplying the former by the corresponding tissue densities of 0.92 (adipose tissue) or 1.1 (lean tissue).

**FIGURE S3 Sit-to-stand test repetitions at baseline and Week 72.**

*Note:* Observed data are mean (standard deviation) from the in-trial period.

## References

1.	Borga M, Ahlgren A, Romu T, Widholm P, Dahlqvist Leinhard O, West J. Reproducibility and repeatability of MRI-based body composition analysis. Magn Reson Med 2020;84:3146-3156.

2.	Borga M, Thomas EL, Romu T, et al. Validation of a fast method for quantification of intra-abdominal and subcutaneous adipose tissue for large-scale human studies. NMR Biomed 2015;28:1747-1753.

3.	Karlsson A, Rosander J, Romu T, et al. Automatic and quantitative assessment of regional muscle volume by multi-atlas segmentation using whole-body water-fat MRI. J Magn Reson Imaging 2015;41:1558-1569.

4.	West J, Romu T, Thorell S, et al. Precision of MRI-based body composition measurements of postmenopausal women. PLoS One 2018;13:e0192495.

5.	Middleton MS, Haufe W, Hooker J, et al. Quantifying abdominal adipose tissue and thigh muscle volume and hepatic proton density fat fraction: repeatability and accuracy of an MR imaging-based, semiautomated analysis method. Radiology 2017;283:438-449.

6.	Mandić M, Rullman E, Widholm P, et al. Automated assessment of regional muscle volume and hypertrophy using MRI. Sci Rep 2020;10:2239.

7.	Karlsson A, Peolsson A, Romu T, et al. The effect on precision and T1 bias comparing two flip angles when estimating muscle fat infiltration using fat-referenced chemical shift-encoded imaging. NMR Biomed 2021;34:e4581.

8.	Niklasson E, Borga M, Dahlqvist Leinhard O, et al. Assessment of anterior thigh muscle size and fat infiltration using single-slice CT imaging versus automated MRI analysis in adults. Br J Radiol 2022;95:20211094.

9.	Linge J, Heymsfield SB, Dahlqvist Leinhard O. On the definition of sarcopenia in the presence of aging and obesity-initial results from UK Biobank. J Gerontol A Biol Sci Med Sci 2020;75:1309-1316.

10.	Borga M. MRI adipose tissue and muscle composition analysis-a review of automation techniques. Br J Radiol 2018;91:20180252.

11.	Borga M, West J, Bell JD, et al. Advanced body composition assessment: from body mass index to body composition profiling. J Investig Med 2018;66:1-9.

12.	Linge J, Petersson M, Forsgren MF, Sanyal AJ, Dahlqvist Leinhard O. Adverse muscle composition predicts all-cause mortality in the UK Biobank imaging study. J Cachexia Sarcopenia Muscle 2021;12:1513-1526.

13.	Sattar N, Neeland IJ, Dahlqvist Leinhard O, et al. Tirzepatide and muscle composition changes in people with type 2 diabetes (SURPASS-3 MRI): a post-hoc analysis of a randomised, open-label, parallel-group, phase 3 trial. Lancet Diabetes Endocrinol 2025;13:482-493.

14.	Linge J, Birkenfeld AL, Neeland IJ. Muscle Mass and Glucagon-Like Peptide-1 Receptor Agonists: Adaptive or Maladaptive Response to Weight Loss? Circulation 2024;150:1288-1298.

15.	Neeland IJ, Marso SP, Ayers CR, et al. Effects of liraglutide on visceral and ectopic fat in adults with overweight and obesity at high cardiovascular risk: a randomised, double-blind, placebo-controlled, clinical trial. Lancet Diabetes Endocrinol 2021;9:595-605.
